# EMS utilization predictors in a Mobile Integrated Health (MIH) program

**DOI:** 10.1186/s12911-021-01409-w

**Published:** 2021-02-04

**Authors:** Luis M. Pinet-Peralta, Lukas J. Glos, Evan Sanna, Brian Frankel, Ernest Lindqvist

**Affiliations:** 1 Maryland Institute for Emergency Medical Services Systems, University of Maryland School of Medicine, Baltimore, USA; 2 The School of Public Policy, University of Maryland Baltimore County, Baltimore, USA; 3 Prince George’s County Fire and EMS Department, Largo, USA

**Keywords:** Service, Emergency health, Health care, Community, Mobile units, Emergency, Program specialist, Population

## Abstract

**Background:**

The provision of unnecessary Emergency Medical Services care remains a challenge throughout the US and contributes to Emergency Department overcrowding, delayed services and lower quality of care. New EMS models of care have shown promise in improving access to health services for patients who do not need urgent care. The goals of this study were (1) to identify factors associated with EMS utilization (911) and (2) their effects on total EMS calls and transports in an MIH program.

**Methods:**

The study sample included 110 MIH patients referred to the program or considered high-users of EMS services between November 2016 and September 2018. The study employed descriptive statistics and Poisson regressions to estimate the effects of covariates on total EMS calls and transports.

**Results:**

The typical enrollee is a 60-year-old single Black male living with two other individuals. He has a PCP, takes 12 medications and is compliant with his treatment. The likelihood of calling and/or being transported by EMS was higher for males, patients at high risk for falls, patients with asthma/COPD, psychiatric or behavioral illnesses, and longer travel times to a PCP. Each prescribed medication increased the risk for EMS calls or transports by 4%. The program achieved clear reductions in 911 calls and transports and savings of more than 140,000 USD in the first month.

**Conclusions:**

This study shows that age, marital status, high fall risk scores, the number of medications, psychiatric/behavioral illness, asthma/COPD, CHF, CVA/stroke and medication compliance may be good predictors of EMS use in an MIH setting. MIH programs can help control utilization of EMS care and reduce both EMS calls and transports.

## Background

For more than twenty years, the demand for Emergency Medical Services (EMS) and Emergency Department (ED) care services in the US has risen consistently, contributing to increasing healthcare costs and impacting quality of care [1]. Some consider a proportion of these services medically unnecessary because they involve low-acuity conditions that are more appropriately handled in settings other than an emergency room [2]. Accurate judgements on medical necessity are complex and require detailed clinical assessments, laboratory tests and medical interventions that challenge even the most seasoned clinicians [3]. Nevertheless, some patients with varying degrees of acuity who seek emergency care can be safely treated by EMS clinicians and/or transported to other non-ED facilities where they can receive definitive care. Medically unnecessary emergency care represents anywhere from 13 to 32% of all EMS calls and cost EMS services an average of 448.50 USD per transport [4–7]. For a jurisdiction responding to 10,000 calls per year, this translates into 1300–3200 transports costing anywhere between 583,050 USD and $1,435,200 USD per year.

Unnecessary EMS care also contributes to overcrowded emergency departments, delays in EMS services and potential reductions in quality of care [4, 8, 9]. Since reimbursement for services occurs only when EMS transports patients to an ED, there is a strong financial incentive to provide services even when they are unnecessary [10]. Furthermore, the current ambulance fee schedule reimburses ambulance suppliers on a Fee-For-Service (FFS) basis, which promotes volume over efficiency [11]. The Centers for Medicare and Medicaid Innovation (CMMI) is implementing a pilot program to explore alternative destinations that can potentially save between 200 and 500 million USD per year [12].

New EMS models of care, finance and delivery have emerged in an effort to control unnecessary EMS care and improve patient outcomes. Some of these models have shown promise in reducing the number of low acuity EMS services and improving access for patients who do not need an ED. Three innovative delivery models include Community Paramedicine (CP), Mobile Integrated Healthcare (MIH) and Alternative Destination (AD) programs. CP programs involve expanded roles for clinicians, while MIH programs involve the use of technology (e.g. telemedicine) and AD programs focus on transporting non-urgent patients to destinations that can appropriately offer definitive treatment to patients (e.g. urgent care centers) other than the ED. All these programs follow patient-centered care approaches, similar to the development of Advanced Primary Care (APC) models.

MIH programs have shown improvements in health-related quality of life as well as reductions in ER transports, and ER and hospital admission and readmission rates [12, 13]. Maryland has six (6) active MIH pilot programs throughout the state. One such program is in Prince George’s County, located in the state of Maryland and bordering with Washington, D.C. Prince George’s (PG) County is the second most populous county in Maryland at 905,161 residents (2017 estimate). The top ten leading causes of death in the county include heart disease, cancer, stroke, injuries, diabetes, septicemia, nephritis and pneumonia. Most of these conditions are chronic and require highly specialized and coordinated care. Although the population has access to five major hospitals, the availability of Primary Care Practitioners (PCP) is less than ideal with one PCP per 1,131 residents [14]. Moreover, a large percentage of the population resides in Health Professional Shortage Areas (HPSAs) for primary care, dental and mental health services [15]. This lack of access to primary care leads many residents to rely on the 911 emergency services system to get the care they need.

### Prince George’s (PG) county MIH program

In response to the many unmet medical, social and behavioral needs of the county, PG Fire and EMS developed a pilot MIH program to improve access to care and reduce high EMS utilization. To understand EMS service utilization trends, PG analyzed 911 calls from July 2015 through June 2016, using electronic patient care report and computer-aided dispatch data. PG defined high utilization as 5 or more 911 calls in any six-month interval, based on guidelines from the Maryland Mobile Integrated Community Health Optional Supplemental Protocol Program, which is consistent with the literature [16]. Using this threshold, PG identified 1,390 patients who requested EMS services 5 or more times and 213 patients who requested EMS services 10 or more times, These High-Frequency (HF) users called EMS more than 8,500 times and requested over 16,400-unit responses in a single year. To be included in the pilot, patients needed to be identified as either HF users based on 911 calls or be considered high-risk by EMS and hospital clinicians, and then referred to the program (Low-Frequency users or LF). High-risk referrals included patients with poorly managed complex chronic illnesses, minimal knowledge of disease processes and/or multiple medications, identified during EMS transport or soon after ED discharge. Also referred were patients receiving care through a Patient-Centered Medical Home (PCMH) program by an established home health care agency; patients that did not give consent were excluded from participation. Once in the program, the MIH team met with patients to perform a Home Safety Assessment (HSA), a Fall Risk Assessment (FRA), a nutrition evaluation, a medication review, physical and mental health assessment, and link patients with appropriate community-based services (intervention). The HSA captured conditions outside and inside patients’ homes considered potentially unsafe (e.g., unstable handrails/stairs, unsecured rugs/furniture), as well as medical (e.g., unsecured oxygen tubing) and general (e.g., untested smoke/CO detectors) risks. The FRA was based on the Hendrich II Fall Risk Model, which provides risk for falls based on gender, mental and emotional status, symptoms of dizziness and medication increasing risk categories (a score of 5 or greater = high risk for falls). The team also used the Healthy Days Core Model (CDC HRQOL-4) and a medication questionnaire tool as part of the assessment process. In 2016, the MIH pilot program was staffed with a paramedic team, and added nurse practitioners, community nurses and social workers in 2017 and 2018, through a collaborative effort between the Prince George’s Fire and EMS Department, and the County Health Department. In contrast with the traditional approach to EMS care where paramedics treat an acute injury/illness and then transport a patient to an ED for further care, this program embeds non-EMS practitioners in the EMS team to address medical, social and behavioral patient needs at the scene without the need to transport, unless the patient requires ED care. This approach improves access to services, facilitates patient engagement and self-efficacy, helps control 911 service utilization/cost and can improve patient outcomes. By September 2018, the program had served a total of 137 patients.

During program enrollment, patients were required to remain in close contact with the MIH team (engagement), who assisted patients with coordination of care, reconnecting them with their PCP or connecting them to a new one. MIH clinicians also facilitated transportation to health appointments and bridged health literacy gaps. During program enrollment, the MIH team also assisted with medication therapy management through a physician or pharmacist, coordinated referrals to specialists (including behavioral health) and addressed social determinants of health. After 4 months of enrollment, all patients underwent a quality assurance review before discharge. Separation occurred either because of patients’ failure to engage, patient dropout, or death. Patients were encouraged to maintain their relationship with the MIH clinicians after discharge and could re-enroll in the program. They also received follow-up from the MIH team at regular intervals after discharge.

Our research goals were to identify (1) the factors associated with EMS utilization and (2) their effects on total EMS calls and transports.

## Methods

### Data and sample

Between November 2016 and September 2018, 137 patients participated in PG’s MIH pilot program. The data collected by the MIH team included EMS (911) calls, transports and dispatches, socio-demographics (e.g. age, race/ethnicity, sex), insurance/access (e.g. private, Medicare, Medicaid), assessments and medications (e.g. fall risk scores, number of medications), as well as data on clinical/chronic illness (e.g. diabetes, hypertension, chronic heart failure).

Twenty-seven patients were excluded from the original dataset because key variables of interest had missing data, including base calls and transports, sociodemographic variables and chronic health conditions. The final sample, therefore, consists of 110 patients of whom 45 requested emergency medical services five or more times before being recruited into the program, and were considered HF users, and 65 patients, who requested emergency medical services less than five times before being recruited into the pilot program, and were considered LF users.

### Statistical analysis

The analysis was conducted in two parts. The first part includes a descriptive analysis of our study sample. Because of our interest in the differences between high- and low-frequency EMS users, the analysis presents proportions for all measures for all participants and by utilization frequency.

The second part includes a regression analysis, in which the outcome variables are the (1) number of 911 calls and (2) the number of 911 transports, and the covariates include socio-demographic, insurance/access, assessments and medications and clinical/chronic illness data. Seventy-three observations had complete data and were therefore included in the regression models. Because the outcome measures represent counts of events, we utilize a Poisson regression model and report incidence rate ratios (IRRs) for each of our covariates. Because of the variability of exposures in EMS calls and transports among patients, we used census population estimates as an offset variable. We also conducted a collinearity test, as well as sensitivity analyses discussed in the results section. All analyses were conducted using Stata 15 statistical package and used a 99% significance level. This study was submitted and considered exempt from IRB review by the University of Maryland Institutional Review Board (HP-00086030). The dataset is available from the corresponding author on reasonable request.

## Results

### Descriptive analysis

Table 1 include descriptive statistics. The analytical file included data on 110 patients. The typical enrollee is a 60-year old single Black male living with two other individuals. He has a PCP and takes him 23 min to get to his/her office, and has Medicare coverage but is not dually eligible. He takes 12 medications for at least one chronic condition and is compliant with his treatment.

**Table 1:** Demographic, insurance and medical characteristics of MIH patients

	All (n = 110)			HF group (n = 45)			LF group (n = 65)			*P*^d^
	*N*	%	*μ*	*N*	%	*μ*	*N*	%	*μ*	
Sociodemographic										
Age (years)										
19–33	12	10.9	60.3	5	11.1	58.2	7	10.8	61.8	0.538
34–48	14	12.7		5	11.1		9	13.8		
49–64	35	31.8		18	40.0		17	26.2		
65–78	34	30.9		13	28.9		21	32.3		
79+	15	13.6		4	8.9		11	16.9		
Race/ethnicity										
White	26	23.6		9	20.0		17	26.2		0.613
Asian	1	0.9		1	2.2		0	0.0		
Blacks	81	73.6		34	75.6		47	72.3		
Hispanic	2	1.8		1	2.2		1	1.5		
Sex										
Male	56	50.9		19	42.2		37	56.9		0.929
Female	54	49.1		26	57.8		28	43.1		
Marital status										
Single	69	67.6		28	66.7		41	68.3		0.873
Married	25	24.5		12	28.6		13	21.7		
Divorced	5	4.9		1	2.4		4	6.7		
Widowed	3	2.9		1	2.4		2	3.3		
Persons living in residence			2.2			2.2			2.2	0.236
Means of first contact										
Door knock/cold call	1	0.9		1	2.3		0	0.0		0.482
Hospital visit	5	4.5		2	4.5		3	4.6		
Phone call	104	94.5		42	95.5		62	95.4		
Assessments and medications										
Fall risk score (n = 87)^a^										
Low	64	73.6	2.5	28	71.8	2.8	36	75.0	2.4	0.645
High	23	26.4		11	28.2		12	25.0		
Compliance with medications										
Never	10	9.1		6	13.3		4	6.2		0.171
Sometimes	35	31.8		17	37.8		18	27.7		
Always	65	59.1		22	48.9		43	66.2		
Number of medications^b^										
1–3	12	11.9	12.3	5	11.1	12.1	7	12.1	12.5	0.973
4–6	8	7.9		4	9.3		4	6.9		
7–9	4	4.0		1	2.3		3	5.2		
10–12	20	19.8		10	23.3		10	17.2		
13–15	18	17.8		7	16.3		11	19.0		
16–18	34	33.7		14	32.6		20	34.5		
19+	5	5.0		2	4.7		3	5.2		
Insurance/access										
*Has a PCP*										
Yes	102	92.7		42	93.3		60	92.3		0.839
No	8	7.3		3	6.7		5	7.7		
*Has insurance*										
Yes	110	100.0		45	100.0		65	100.0		
No	0	0.0		0	0.0		0	0.0		
*Dual eligible?*										
Yes	18	16.4		10	22.2		8	12.3		0.167
No	92	83.6		35	77.8		57	87.7		
*Primary insurance*										
Private	14	12.7		4	8.9		10	15.4		0.439
Medicaid	35	31.8		17	37.8		18	27.7		
Medicare	61	55.5		24	53.3		37	56.9		
*Travel times to PCP*										
0–9 min	9	8.2	22.8	4	8.9	22.4	5	7.7	23.1	0.989
10–19 min	34	30.9		15	33.3		19	29.2		
20–29 min	41	37.3		16	35.6		25	38.5		
30–39 min	16	14.5		6	13.3		10	15.4		
40 min or longer	10	9.1		4	8.9		6	9.2		
Clinical/chronic illness										
*Asthma/COPD*										
No	73	*66.4*		29	*64.4*		44	67.7		0.723
Yes	37	*33.6*		16	35.6		21	32.3		
*Hypertension*										
No	35	31.8		16	35.6		19	29.2		0.484
Yes	75	68.2		29	64.4		46	70.8		
*Hypercholesterolemia*										
No	81	73.6		32	71.1		49	75.4		0.617
Yes	29	26.4		13	28.9		16	24.6		
*Chronic heart failure*										
No	84	76.4		31	68.9		53	81.5		0.125
Yes	26	23.6		14	31.1		12	18.5		
*Stroke/CVA*										
No	85	77.3		35	77.8		50	76.9		0.916
Yes	25	22.7		10	22.2		15	23.1		
*Psychiatric/behavioral*										
No	70	63.6		25	55.6		45	69.2		0.143
Yes	40	36.4		20	44.4		20	30.8		
*Diabetes*										
No	64	58.2		28	62.2		36	55.4		0.475
Yes	46	41.8		17	37.8		29	44.6		

*HF* High frequency users, *LF* low frequency users

^a^Includes only patients who were considered at risk for falls

^b^Includes only patients with reported number of medications

^c^Eight (8) observations with “No response”

^d^Significance = .05

Eighty-four (76%) patients were 49 years or older and 81 (74%) were Black, with an almost even split between men and women. Sixty-nine were single (68%), 25 (25%) were married and 104 (95%) were introduced to MIH via phone call. Sixty-five (74%) had low fall risk scores with an average score of 2.5. One-hundred (91%) were sometimes or always compliant with their medication regimens and took an average of 12 medications, with 77 (76%) of them taking 10 or more medications. The majority (102, 93%) have a PCP and all 110 patients have insurance, with Medicare, Medicaid and private insurance covering 61 (56%), 35 (32%) and 14 (13%) patients, respectively. Patients took an average of 23 min to get to their PCP, with 101 (82%) taking anywhere from 10 to 39 min. Seventy-five (68%) patients had hypertension followed by 46 (42%) with diabetes, 40 (36%) with psychiatric/behavioral disorders, 37 (34%) with asthma/COPD, 29 (26%) with hypercholesterolemia, 26 (24%) with Chronic Heart Failure (CHF) and 25 (23%) have had a stroke/CVA.

From the 110 patients in the full sample, 45 (41%) were classified as High-Frequency (HF) users, with the other 65 (69%) classified as Low-Frequency (LF) users of EMS services (Tables 1 and 2). The HF group was relatively younger, 58 years on average, and had a larger proportion of women (26, 58%). They also had slightly higher fall risk scores (2.8) and were less compliant with medications. In terms of access to care, the rates for PCP were higher on the HF group, where 42 (93%) had a PCP compared with 60 (92%) on the LF group. Dual eligibility was higher on the HF group with 10 (22%) patients, compared with the LF group where only eight (12%) patients. The HF group had lower rates of private insurance and a slightly larger share of Medicaid beneficiaries. Travel times to PCPs were similar between groups. Asthma/COPD and hypercholesterolemia were slightly more prevalent in the HF group, although they had higher rates of CHF and psychiatric/behavioral illness. Hypertension and diabetes were not as prevalent in the HF group as with the LF group.

**Table 2:** Poisson regression, MIH program (n = 73)

911 Calls	IRR	Std. Err	z	P >|z|	95% CI	
*Age category*						
19–33 (ref)						
34–48 Years	1.119	0.272	0.46	0.644	0.695	1.802
49–64 Years	0.887	0.207	− 0.51	0.608	0.561	1.402
65–78 Years**	0.514	0.121	− 2.83	0.005	0.324	0.815
79 + ***	0.080	0.032	− 6.4	0.000	0.037	0.173
*Marital status*						
Single (ref)						
Married***	0.358	0.061	− 6.06	0.000	0.257	0.499
Divorced***	0.495	0.102	− 3.42	0.001	0.331	0.740
Widowed**	4.097	1.943	2.97	0.003	1.617	10.381
*Sex*						
Female (ref)						
Male***	2.067	0.230	6.52	0.000	1.662	2.572
*Race*						
White (ref)						
Asian	0.000	0.005	− 0.02	0.983	0.000	
Black***	1.896	0.327	3.71	0.000	1.352	2.660
Hispanic***	7.652	2.208	7.05	0.000	4.347	13.472
*Fall risk category*						
High risk***	2.795	0.389	7.39	0.000	2.128	3.671
*Persons living in the residence*						
1 (ref)						
2	0.989	0.142	− 0.08	0.939	0.746	1.311
3***	2.467	0.463	4.81	0.000	1.708	3.564
4**	2.380	0.831	2.49	0.013	1.201	4.717
6***	2.642	0.746	3.44	0.001	1.519	4.594
7	0.503	0.282	− 1.22	0.221	0.167	1.512
*Asthma/COPD*						
Yes***	2.693	0.398	6.71	0.000	2.016	3.598
*Hypertension*						
Yes**	1.482	0.237	2.45	0.014	1.082	2.028
*High cholesterol*						
Yes	0.774	0.111	− 1.79	0.073	0.584	1.025
*CHF*						
Yes**	0.682	0.093	− 2.82	0.005	0.523	0.890
*CVA/Stroke*						
Yes**	1.499	0.198	3.06	0.002	1.157	1.943
*Psychiatric and/or Behavioral*						
Yes***	1.580	0.195	3.71	0.000	1.241	2.011
*Diabetes*						
Yes	1.100	0.128	0.81	0.416	0.875	1.382
*Compliance with medications*						
Never (ref)						
Sometimes	1.258	0.253	1.14	0.255	0.847	1.867
Always	1.236	0.212	1.23	0.218	0.882	1.731
Number of medications***	1.044	0.010	4.42	0.000	1.024	1.064
*Primary insurance*						
Private (ref)						
Medicaid***	0.297	0.067	− 5.42	0.000	0.191	0.461
Medicare	1.101	0.184	0.57	0.566	0.793	1.529
*Travel times*						
0–9 min (ref)						
10–19 min***	5.793	1.502	6.78	0.000	3.485	9.628
20–29 min***	2.837	0.765	3.87	0.000	1.673	4.812
30–39 min***	17.027	5.595	8.63	0.000	8.942	32.421
40 min and longer***	10.200	3.303	7.17	0.000	5.407	19.243
*Dual eligible*						
Yes***	0.511	0.089	− 3.87	0.000	0.363	0.717
*_cons*	0.000	0.000	− 35.49	0.000	0.000	0.000
*logPop*	1	(offset)				

*Significance at p < .05; **significance at p < .01; ***significance at p < ..001

Since a large number of MIH patients (65) were not HF users of EMS services, non-parametric statistics were run to determine if there were any associations between covariates in the HF and LF groups that could influence 911 calls and transports counts. Based on a 95% significance level, the study found no statistically significant associations between sociodemographic, assessment & medications, insurance/access to care or clinical/chronic illness covariates and eligibility, with only moderate, not statistically significant associations between persons living in residence, compliance with medications, dual eligibility, CHF and psychiatric/behavioral illnesses. Given these results, it was appropriate to use the full sample to test for predictors of EMS calls and transports. Prior to running the regressions, collinearity tests revealed no significant correlations between covariates.

### Regression models

Tables 2 and 3 include the regression results for EMS calls and transports. For EMS calls, patients ages 65 and older were less likely to call EMS compared to their younger counterparts. Patients ages 64–78 were 49% (p 0.005) less likely to call EMS, whereas those 79 years and older were 92% (p = 0.000) less likely to call EMS, compared to the youngest group. Patients who were either married or divorced were 65% (p = 0.000) and 51% (p = 0.001) less likely to call EMS, respectively, compared to single patients. In contrast, widowed patients were more than 4 times more likely to call EMS (p = 0.003). Men were two (2) times (p = 0.000) more likely to call EMS compared to women. In terms of race, Blacks had 89% (p = 0.000) higher chance to call EMS and Hispanics showed a sevenfold increase (p = 0.000) in their chance of calling EMS, when compared to White patients. Patients with a high fall risk score were 2.7 times (p = 0.000) more likely to call EMS compared to patients with low scores. Patients living with three to six additional people in the same residence had more than twice the chance of calling EMS compared to patients living with one person.

**Table 3:** Poisson regression, MIH program (n = 73)

911 Transports	IRR	Std. Err	z	P >|z|	95% CI	
*Age category*						
19–33 (ref)						
34–48 Years	1.374	0.386	1.13	0.258	0.793	2.382
49–64 Years	0.548	0.150	− 2.19	0.028	0.320	0.938
65–78 Years	0.587	0.157	− 2.00	0.046	0.348	0.990
79 + ***	0.094	0.047	− 4.74	0.000	0.035	0.249
*Marital status*						
Single (ref)						
Married***	0.286	0.068	− 5.30	0.000	0.180	0.454
Divorced**	0.441	0.120	− 3.02	0.003	0.259	0.750
Widowed***	10.206	5.675	4.18	0.000	3.432	30.351
*Sex*						
Female (ref)						
Male***	2.412	0.335	6.33	0.000	1.837	3.168
*Race*						
White (ref)						
Asian	0.000	0.020	− 0.01	0.988	0.000	
Black**	1.998	0.458	3.02	0.002	1.276	3.131
Hispanic***	3.675	1.353	3.53	0.000	1.785	7.564
*Fall risk category*						
High risk	1.700	0.316	2.86	0.004	1.181	2.447
*Persons living in the residence*						
1 (ref)						
2	0.966	0.174	− 0.19	0.846	0.678	1.375
3*	1.620	0.377	2.08	0.038	1.027	2.556
4**	3.509	1.555	2.83	0.005	1.472	8.365
6***	4.903	1.668	4.67	0.000	2.517	9.552
7	2.105	1.268	1.24	0.217	0.646	6.855
*Asthma/COPD*						
Yes***	4.346	0.820	7.78	0.000	3.002	6.291
*Hypertension*						
Yes	0.840	0.182	− 0.81	0.419	0.549	1.284
*High cholesterol*						
Yes**	1.749	0.346	2.83	0.005	1.187	2.578
*CHF*						
Yes***	0.454	0.081	− 4.42	0.000	0.320	0.644
*CVA/Stroke*						
Yes*	1.443	0.262	2.02	0.044	1.010	2.060
*Psychiatric and/or Behavioral*						
Yes***	1.833	0.298	3.73	0.000	1.333	2.522
*Diabetes*						
Yes***	1.940	0.274	4.69	0.000	1.471	2.560
*Compliance with medications*						
Never (ref)						
Sometimes**	1.949	0.494	2.63	0.008	1.186	3.203
Always	1.505	0.332	1.85	0.064	0.977	2.320
Number of medications***	1.045	0.013	3.68	0.000	1.021	1.071
*Primary insurance*						
Private (ref)						
Medicaid	0.599	0.179	− 1.72	0.086	0.334	1.075
Medicare**	2.192	0.554	3.11	0.002	1.336	3.597
*Travel times*						
0–9 min (ref)						
10–19 min***	5.934	1.684	6.27	0.000	3.402	10.350
20–29 min***	3.669	1.082	4.41	0.000	2.059	6.539
30–39 min***	8.748	3.275	5.79	0.000	4.200	18.221
40 min and longer***	15.094	5.664	7.23	0.000	7.234	31.495
*Dual eligible*						
Yes***	0.295	0.072	− 5.03	0.000	0.184	0.475
*_cons*	0.000	0.000	− 30.29	0.000	0.000	0.000
*logPop*	1	(offset)				

*Significance at p < .05; ** significance at p < .01; *** significance at p < .001

Patients with asthma/COPD were 2.7 (p = 0.000) times more likely to call EMS, and those with hypertension, CVA/Stroke, and psychiatric or behavioral conditions had between 48 and 58% chance of calling EMS compared with patients without these illnesses. Patients with high cholesterol were 33% less likely to call EMS and those with diabetes were 10% more likely to call EMS compared with patients without these illnesses, but the results were not statistically significant. Compliance with medications showed an increased risk for calling EMS, but the results were not statistically significant. For each medication patients took, the changes of calling EMS increased by 4% (p = 0.000). Patients covered through Medicaid were 71% (p = 0.000) less likely to call EMS compared with patients with private insurance coverage, whereas patients eligible for dual coverage were 49% (p = 0.000) less likely to call EMS. Travel times to PCP offices showed large and statistically significant results. Patients with travel times greater than 30 min were between 10 and 17 times (p = 0.000) more likely to call 911.

Patients 49–64 and 65–78 years were 46% (p = 0.02) and 48% (p = 0.04) less likely to be transported compared to those ages 19–33, whereas patients 79 and older were 91% (p = 0.000) less likely to be transported compared to the youngest group. Marital status had a similar effect as for 911 calls. Married patients were 72% (p = 0.000) less likely to require transport compared to single patients. Divorced patients also showed a protective effect, with 56% (p = 0.003) less chance for transport. In contrast, widowed patients were 10 times (p = 0.000) more likely to require transport compared with single patients.

Males were 2.4 times (p = 0.000) more likely to be transported compared to women. Blacks and Hispanics were 1.9 (p = 0.002) and 3.6 (p = 0.000) times more likely to require transport, respectively, compared with Whites. Living with three to six people in the same residence increased the chances of transport anywhere between 1.6 and 4.9 times, compared with living with one person only.

Chronic illnesses differed somehow between transports and calls. Patients with diabetes, psychiatric or behavioral illnesses, CVA/Stroke, high cholesterol and asthma/COPD were more likely to require transport compared with patients without any of these conditions. Asthma/COPD, psychiatric/behavioral conditions and diabetes had the largest effect, with 4.3 (p = 0.000), 1.8 (p = 0.000) and 1.9 (p = 0.000) higher chances of transport, respectively, compared with patients without these conditions. Patients who were sometimes compliant with their medications were 50% (p = 0.008) more likely to require transport compared with patients who were never compliant. The effect on the number of medications was similar as with 911 calls, with a 4% (p = 0.000) chance of transport for each medication. Patients with Medicare coverage were 2.1 times (p = 0.002) more likely to require transport compared with those with private insurance. Travel times for 911 transports were significant and showed increased likelihood of transport by up to 15 times (p = 0.000) when travel exceeded 40 min or longer, compared to travel times shorter than 10 min.

### Sensitivity analysis

After dropping non-significant variables from the model, the likelihood of EMS calls remained for patients who were married and widowed, with a high fall risk score, patients who referred CVA, psychiatric or behavioral illness, CHF and asthma/COPD, the number of prescription medications, and travel times. For EMS transports, the effects remained for married or widowed male patients, those with asthma/COPD, CHF, psychiatric/behavioral illnesses, the number of prescription medications and travel times.

### 911 call and transport data

**Fig. 1:**
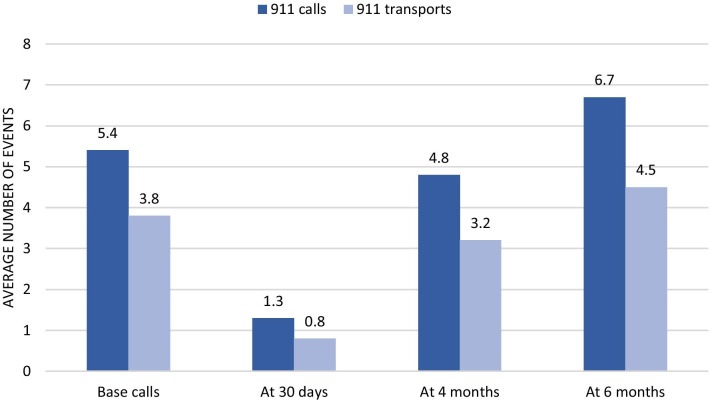
Average EMS calls and transports before and after first MIH visit, 2016–2018

Participants called 911 a total of 630 times and requested 412 transports before the study (baseline). From these, 506 (80%) calls and 326 (79%) transports came from the HF group. After the first 30 days of pilot implementation, there were a total of 163 calls and 95 transports, a reduction of 74% and 77%, respectively. After four months, the reduction in calls and transports was only 10% and 13%, respectively, compared with the baseline. As shown in Table 4 and Fig. 1, both calls and transports experienced sharp reductions 30 days after the first patient visit by the MIH team. At the 4th month mark, calls and transports remained below baseline although both increased compared to the 30-day mark. By the 6th month mark, both call and transports showed a marked increase compared with the baseline.

**Table 4:** Average 911 calls and transports before and after first MIH visit, 2016–2018

	Total sample (110)	HF group (n = 45)	LF group (n = 65)
	*N*	*N*	*N*
*Call data*			
Base calls	5.4	9.7	2.5
At 30 days	1.3	2.4	1.9
At 4 months	4.8	8.2	2.6
At 6 months	6.7	10.4	4.1
*Transport data*			
Base transports	3.8	6.6	1.9
At 30 days	0.8	1.4	0.4
At 4 months	3.2	5.2	1.8
At 6 months	4.5	6.3	3.2

*HF* High frequency users, *LF* low frequency users

## Discussion

Some of the predictors for EMS use in general may apply differently to populations targeted by MIH programs, including age. Older patients had a much lower chance of being transported compared to younger ones, although they may still seek EMS care more frequently [17]. Marital status is a well-known predictor of health, where unmarried individuals report poorer health and higher risks for morbidity and mortality compared to married ones, and our data is consistent with the literature. Interestingly, widowed patients showed a high likelihood for both 911 calls and transports, even after model adjustments.

Patients with high fall risk scores were more likely to call EMS and need emergency transport as a result, so this may be a reliable predictor for both outcomes of interest. It is unclear why the number of people living with the patient has a positive effect on EMS calls and transports, although the results were not very consistent. One explanation may be that when people live and know the patient well, they may be better at recognizing the need to both call EMS and to encourage patients to go to a hospital.

Chronic illnesses, particularly diabetes, psychiatric or behavioral illnesses, CHF and asthma/COPD, were consistent predictors of both EMS calls and transports. These results are not surprising, given that patients with chronic conditions experience more frequent acute disease exacerbations that regularly need emergency care. Another consistent predictor was the number of medications patients took, increasing by approximately 4% for each medication patients take. This means that patients taking 12 medications can see their risk for EMS calls and transports increasing by 48%. Compliance with medications showed a statistically significant likelihood for EMS transports but was not significant for EMS calls, although it also showed positive effects. This may be because patients who are managing their conditions more closely and pay close attention to their medication regimens, may be more acutely aware when their condition deteriorates and when they truly need to get care.

Patients covered through Medicaid had a lower likelihood for EMS calls (71%) and those with Medicare had a high likelihood for transports (2.1 times) across the models compared with private insurance. Medicaid patients may have experienced greater financial barriers to care compared with Medicare or private insurance patients and requesting fewer 911 services, but present with higher acuity of illness when calling 911, although this was not something we could capture with the data. These results are also consistent with ED use by insurance type, where Medicare represents 87% of all ED visits for patients 65 and older and shares 16% among those ages 45 through 64 [18].

When EMS receives a call, the information comes almost exclusively from the people at the scene (e.g., patients), who provide dispatchers with information they use to make a determination on whether or not to send an ambulance. This information may not be as accurate or relevant as the information obtained by EMS clinicians at the scene, where a series of more objective assessments (e.g. vital signs, medications, fall risk scores) help construct a decision for transport. This dynamic may be a reason why the transport results remained more stable and consistent compared with the calls results.

The program achieved clear reductions of both EMS calls and transports during the four-month intervention period, translating into savings of more than 142,016 USD of avoidable EMS transports during the first month alone, based on average transport costs. Nevertheless, these reductions showed a diminished rate of return throughout the pilot program and ended close to baseline by the time patients graduated from the program. These short-lived effects may be due, in part, to the complexity of the patients’ chronic conditions and the need for more sustained multi-disciplinary teams to support patients’ medical, behavioral and social needs. Given that the intervention ended at the 4th month, patients may have lost their ability to travel to their PCP for follow-up care, may not have had time to develop the self-efficacy necessary to manage their chronic illnesses or their multiple medications or may have experienced a greater degree of social isolation, which may have influenced their use of 911 services [19]. Most patients in our sample had access to a PCP and had public insurance coverage. However, we did not have information about the quality of the patient-provider relationship, the frequency of visits or the degree of care coordination between their PCP and other specialists needed to help patients manage their conditions.

## Limitations

One limitation of our study is the small sample size, given there were more than 1300 patients considered to be high-users but only 137 (10%) agreed to participate. Another limitation is selection bias, since patients who were either high-users or referred to the program and agreed to participate may be systematically different from patients who chose not to participate in the program. Our data are also limited to what PG collected during the program and may not be capturing factors or exposures that are important and significant in terms of EMS utilization, such as education or income. Finally, the length of the MIH intervention is another limitation. The efforts to address the types of chronic illnesses, comorbidities and risk factors prevalent in the target population require long-term, collaborative and multidisciplinary approaches and interventions. Moreover, these interventions must address the broader social determinants of health, including health behaviors, rather than focus on health determinants alone.

## Conclusions

This study showed that age, marital status, sex, fall risk, the number of medications, psychiatric/behavioral illness, asthma/COPD, CHF, stroke and medication compliance may be good predictors of EMS use in an MIH setting. The reduction of EMS calls and transports during the intervention period indicates that an MIH program can be effective in managing utilization of emergency services. Appropriate support for these programs, including operational, financial and political support, will determine their sustainability and long-term success.

## Data Availability

The dataset used for this study is available from the corresponding author on reasonable request.

## Abbreviations

EMS: Emergency Medical Services
ED: Emergency Department
CMMI: Centers for Medicare and Medicaid Innovation
FFS: Fee-For-Service
MIH: Mobile Integrated Health
CP: Community Paramedicine
AD: Alternate Destination
PG: Prince George’s
PCP: Primary Care Practitioner
HF: High Frequency
LF: Low Frequency
PCMH: Patient-Centered Medical Home
HAS: Home Safety Assessment
FRA: Fall Risk Assessment
IRR: Incidence Rate Ratio
HPSA: Health Professional Shortage Area
